# Influence of Social and Demographic Factors on the Montreal Cognitive Assessment (MoCA) Test in Rural Population of North-Eastern Greece

**DOI:** 10.3390/geriatrics6020043

**Published:** 2021-04-17

**Authors:** Anna Tsiakiri, Konstantinos Vadikolias, Grigorios Tripsianis, Pinelopi Vlotinou, Aspasia Serdari, Aikaterini Terzoudi, Ioannis Heliopoulos

**Affiliations:** 1 Department of Neurology, Medical School, Democritus University of Thrace, Univeristy Hospital of Alexandroupolis, 68100 Dragana, Greece; kvadikol@med.duth.gr (K.V.); pinevlot@med.duth.gr (P.V.); terzoudi@med.duth.gr (A.T.); iiliop@med.duth.gr (I.H.); 2 Laboratory of Medical Statistics, Medical School, Democritus University of Thrace, 68100 Alexandroupolis, Greece; gtryps@med.duth.gr; 3 Department of Child & Adolescent Psychiatry, Medical School, Democritus University of Thrace, University Hospital of Alexandroupolis, 68100 Dragana, Greece; aserntar@med.duth.gr

**Keywords:** MoCA, mild cognitive impairment, healthy adults, dementia, rural population, neuropsychology

## Abstract

The current study aims to investigate the influence of socio-demographic factors on the Montreal Cognitive Assessment (MoCA) test results in a Greek-speaking population consisting of a sample of healthy older adults, individuals with mild cognitive impairment (MCI), and dementia patients in rural areas. In addition, the current research focuses on determining optimal cut-off scores for the clinical diagnoses of MCI and dementia. The data originated from 283 participants in an ongoing registry of the Neurology Department of Alexandroupolis University Hospital, recruited in different rural districts of north-eastern Greece, across a broad range of educational and occupational categories. Total and sub-domain scores for the MoCA varied significantly, according to sex, age, and education, among the three study groups. The optimal cut-off points of 25/26 for the MoCA total score was determined to classify healthy subjects from individuals with MCI, 24 to discriminate healthy participants from demented, and 21/22 to discriminate subjects with MCI from dementia. Overall, the clinical use of the MoCA test can be supported by demographically adjusted standard scores in a Greek-speaking rural population. These findings serve to improve the diagnostic accuracy and utility of the MoCA test.

## 1. Introduction

Performance in various cognitive domains of neuropsychological functioning can contribute to the diagnosis of dementia and to predicting the progression from mild cognitive impairment (MCI) to dementia [1]. Brief cognitive screening tests for this clinical population are administered at primary health services, in order to maximize early detection. Such tools must be relatively easy to use, quick to administer, and accurate, to ensure their feasibility of use in primary care.

The two most commonly used brief cognitive tests are the Mini Mental State Examination (MMSE) and the Montreal Cognitive Assessment (MoCA). The MMSE is the most commonly used cognitive evaluation tool, which has high levels of sensitivity and specificity for detecting dementia [2]. It consists of 11 items, with maximum score of 30, taking at most 10 min by a trained interviewer [3]. There are many data on its properties in different populations, validating the effects of age, ethnicity, and level of education [4]. A weak point is its lack of sensitivity in detecting dementia at early stages [5,6]; moreover, it does not examine executive functions and there are few tasks for assessing episodic and semantic memory. Individuals with MCI may subsequently develop AD and, yet, score within the normal range on the MMSE [7].

The MoCA appears to be one of the few instruments precisely designed to detect MCI [8]. It is a 30-point scale, including language, short-term memory, visuospatial abilities, attention, and working memory, as well as aspects of executive functioning, language, and orientation. It focuses on tasks associated to frontal lobe executive functioning and draws more attention than the MMSE, which may make it more sensitive in detecting non-AD dementia. Several studies have reported levels of sensitivity and specificity for the detection of MCI and combined MCI/AD using cut-off scores between 23–26 [8,9,10,11,12]. It has also been determined that one point should be added to the total score, in order to correct for the influence of education for individuals with 12 or fewer years of formal education. In Table 1 there is a review in literature about proposed normative values and the diagnostic ability of the MoCA test from 2008 till nowadays.

To date, two studies have used the MoCA in Greek populations. In the first one, conducted in 2016 [13], the researchers focused on healthy adults (aged 20–85 years) and Parkinsonian patients. They concluded that the administration of the MoCA is affected by age and education. The sample of the study consisted of participants in urban centers. The second study, in 2019 [14], dealt with individuals who mentioned subjective cognitive complaints, people with MCI, and demented patients. The participants of this research were adults over 60 years of age from the urban city of Thessaloniki, a city in Northern Greece. Their findings suggested that the MoCA is not affected by age or sex, but is affected by the educational level.

Therefore, there is a need for further normative data of the MoCA for healthy older adults and for residents of rural areas in Greece. It should be taken into account that several items may be culturally biased, which implies the strong need to employ population-based norms. The aforementioned studies have indicated a need for further investigation on such norms and their relationships with other tests, including the MMSE.

The aim of the present study was to investigate the influence of socio-demographic factors on the performance, as well as the interpretation of, the MoCA test results in a Greek-speaking population in a sample of healthy older adults, individuals with MCI, and demented patients in rural areas. The current research was also focused on determining optimal cut-off scores for the clinical diagnoses of MCI and dementia. Moreover, we attempt to provide normative data about the MoCA in the specific sample used.

## 2. Materials and Methods

### 2.1. Subjects

Data from 283 participants in an ongoing registry of the Neurology Department of the University Hospital of Alexandroupolis were used for this analysis. Participants were recruited in different rural districts of north-eastern Greece and in a broad range of categories, according to their education and occupation. The final sample consisted of three (3) groups: (a) Community-dwelling older healthy adults, (b) individuals with MCI, and(c) demented patients. The first group entered the study on the occasion of informative activities conducted by the outpatient dementia clinic of the Neurology Department in rural areas of the district of Evros. The other two groups consisted of participants who visited the outpatient dementia clinic and underwent the MoCA examination as a part of their routine neuropsychological assessment.

The final sample was divided into three age groups (50–64, 65–74, and >74 years) and three educational categories (low: 1–6 years, medium: 7–12 years, and high: more than 13 years; see Table 2).

All participants signed an informed consent form prior to their participation. Approval was also required for the demented patients by their caregiver and/or by a legal representative. Approval for the study was granted by the Ethics Committee of the University Hospital of Alexandroupolis.

### 2.2. Inclusion/Exclusion Criteria

The interview collected biographical information and medical data, including information regarding any medical diagnosis; history of cardiovascular, metabolic, and neurological syndromes; and history of affective diseases. Individuals with MCI and dementia patients underwent a neurological examination, neuropsychological assessment, neuroimaging, and specific biochemical and hematological control.

#### 2.2.1. Healthy Adults

The final normative sample included 64 healthy adults over 50 years old, who scored MMSE >26, according to the latest cut-off scores. Other inclusion criteria were (a) No current or past history of neurological diseases; (b) absence of affective diseases, as verified by Geriatric Depression Scale (GDS) <6 and Neuropsychiatric Inventory (NPI); and (c) absence of medical condition or medication that could affect neuropsychological testing.

#### 2.2.2. Individuals with Mild Cognitive Impairment (MCI)

A total of 100 individuals met the diagnostic criteria for the diagnosis of MCI, as proposed by Petersen et al. [32] and codified in the DSM-V [33]: (a) subjective impression of decline in cognitive functioning, including impressions by the patient, by a family member or other informant, or the clinician; (b) cognitive impairment for the age, shown by formal neuropsychological testing; (c) evidence of the gradual decline on objective cognitive tasks—greater than expected for the age—without falling into the dementia range; (d) preserved general cognitive and daily function; and (e) absence of a prior diagnosis of dementia and other mental condition (e.g., depression, delirium, intoxication, or psychosis) that could account for the observed impairment.

Additional inclusion criteria were (a) age over 50 years; (b) MMSE total score < 26; (c) absence of neurological and cardiovascular diseases; and (d) not under pharmacological treatment with cholinesterase inhibitors, antipsychotic, and/or anticholinergic drugs.

#### 2.2.3. Individuals with Dementia

According to the fifth edition of the Diagnostic and Statistical Manual of Mental Disorders (DSM-5) [34,35], dementia is classified as a “major neurocognitive disorder” (NCD) and meets specific diagnostic criteria: (a) Impairment in only one cognitive domain is enough to qualify for a diagnosis, except for the case of major NCD due to Alzheimer’s disease, where two domains are also required, one of which must be memory impairment; (b) deficits in six cognitive domains (complex attention, executive function, learning and memory, language, perceptual motor, and social cognition), which interfere with independence in everyday activities; (c) cognitive decline based on both subjective concern or an informant and/or a clinician, and the objective demonstration of substantial impairment in cognitive performance on an objective measure; and (d) performance in neuropsychological testing typically falling 2 or more standard deviations (SD) below the normative mean.

In the current study, 119 patients fulfilled the criteria for diagnosis of Major NCD, describing the condition of dementia of any etiology. Participants were excluded if they had (a) a past history of neurological diseases or (b) severe neuropsychiatric symptoms. Participants with unstable medical illness and uncorrected visual or hearing impairment were excluded from all categories (healthy, MCI, dementia).

### 2.3. Procedure

All participants underwent a battery of neuropsychological testing, comprised of the Greek version of the MMSE, the 15-item GDS questionnaire [36], and the Neuropsychiatric Inventory (NPI) [37], in order to detect mood disorders. For the MoCA test, the official Greek translation was used (Version 7; http://www.mocatest.org, accessed on 20 March 2020). The cognitive domains assessed through the 12 sub-tasks werevisuospatial/executive, naming, memory, attention, language, abstraction, delayed recall, and orientation. The original version provides an extra point for individuals with lower education (i.e., ≤12 years). A one-point correction was applied to low and medium education groups. The total score ranges from 0 (worst performance) to 30 (best performance). The interviews took place in a quiet room and every participant was tested individually by the neuropsychologist of the Neurology Department.

### 2.4. Statistical Analysis

Data were analyzed using the Statistical Package for the Social Sciences (SPSS) software, version 19.0 (IBM Corp., Armonk, NY, USA). The normality of quantitative variables was tested by the Kolmogorov–Smirnov test. All quantitative variables are expressed as mean value ± standard deviation (SD), while qualitative variables are expressed as frequencies and percentages (%). To assess the differences in demographic and clinical characteristics between healthy subjects, individuals with MCI, and demented patients, chi-square test and analysis of variance (ANOVA) were used, while post-hoc comparisons were performed using Tukey’s test. Three multivariate analyses of variance (MANOVAs), with a 2 (gender: male, female) × 3 (age: 50–64 years, 65–74 years, >74 years) × 3 (education: low, medium, high) design were conducted, in order to investigate the effects of gender, age, and education on the sub-scale scores of MoCA within each group. Post-hoc comparisons were performed using Sidak’s test. Effect sizes were assessed using partial eta square (η^2^). Value of η^2^ = 0.01 expresses small effect size, 0.06 medium effect size and 0.14 large effect size [38]. The following assumptions of MANOVA were met in our study: Multivariate outliers (using Mahalanobis distance), linearity of the data (using scatter plots), multicollinearity (using Pearson’s r correlation coefficient), homogeneity of covariance matrices (using Levene’s test), and homogeneity of variance–covariance matrices (using Box’s M test). The assumption of an adequate sample size (i.e., the need to have more cases in each group than the number of dependent variables) applied in all groups, although only marginally in the group of healthy subjects. Receiver operating characteristic (ROC) analysis was performed, in order to evaluate the ability of the total MoCA score to discriminate healthy subjects, individuals with MCI, and demented patients. The area under the ROC curve (AUC), sensitivity, specificity, positive and negative predictive values, and positive and negative likelihood ratios were calculated. The optimal cutoff values were derived according to the Youden Index. All tests were two-tailed and statistical significance was considered for *p* values < 0.05.

## 3. Results

### 3.1. Comparison between the Three Groups of Subjects

The demographic and clinical characteristics of healthy subjects, individuals with MCI, and demented patients are given in Table 2. Male sex was more frequent in demented patients (*p* = 0.016). Moreover, demented patients were older (*p* < 0.001) and less educated (*p* < 0.001) compared to healthy subjects and individuals with MCI. One-way ANOVA showed statistically significant differences of MMSE, total MoCA score, and MoCA sub-test scores between the three groups of subjects (all *p* < 0.001); post hoc analysis using Tukey’s test showed that demented patients had lower scores, compared to healthy subjects (all *p* < 0.001) and subjects with MCI (all *p* < 0.001). Furthermore, subjects with MCI had lower scores, compared to healthy subjects (all *p* < 0.05); with the exception of naming (*p* = 0.83), memory (*p* = 0.67), and orientation (*p* = 0.91). All of the above associations remained unchanged, even after adjustment for sex, age, and education level.

### 3.2. Montreal Cognitive Assessment (MoCA)Scoresin Healthy Adults

A total of 64 healthy individuals (14 males, 21.9%), with a mean age of 66.73 ± 7.60 years and mean duration of education of 10.25 ± 4.40 years, were tested. The mean MoCA score of the healthy subjects was 27.58 ± 1.26 and the mean MMSE score was 29.11 ± 0.69. The mean total MoCA score and its sub-category scores, according to the sex, age, and education of subjects, are shown in Table 3. The MoCA total score was not affected significantly by sex (F_1,54_ = 0.026, *p* = 0.87, η^2^ = 0.000), age (F_2,54_ = 0.147, *p* = 0.86, η^2^ = 0.005), or education (F_2,54_ = 0.350, *p* = 0.71, η^2^ = 0.013). MANOVA, which was also used to investigate the effects of sex, age, and education on the sub-scale scores of the MoCA, showed that sex had a statistically significant effect on the sub-score of delayed recall (F_1,54_ = 6.150, *p* = 0.016, η^2^ = 0.102), age had a significant effect on the sub-score of visuospatial (F_2,54_ = 3.568, *p* = 0.035, η^2^ = 0.117), and education had significant effects on the sub-scores of language (F_2,54_ = 4.265, *p* = 0.019, η^2^ = 0.136) and abstraction (F_2,54_ = 4.201, *p* = 0.020, η^2^ = 0.135). In particular, younger adults performed better in the visuospatial ability compared to older participants (*p* = 0.030) but not compared to adults aged 65–74 years (*p* = 0.81); no significant difference was found between ages 65–74 years and >74 years (*p* = 0.81). Furthermore, higher level of education performed better from those with low (*p* < 0.001 and *p* = 0.006, respectively) but not with medium (*p* = 0.05 and *p* = 0.58, respectively) level in the cognitive abilities of language and abstraction. No significant differences in the cognitive abilities of language and abstraction between healthy participants with low and medium level of education were observed (*p* = 0.18 and *p* = 0.11, respectively).

The MoCA score, before applying the one point correction for low and medium education groups, was 26.65 ± 1.37 (low education), 26.25 ± 1.41 (medium education), 27.81 ± 0.93 (high education), *p* < 0.001. Higher level of education performed better MoCA total score than those with low (*p* = 0.01) and medium (*p* = 0.001) level; no significant difference was observed between low and medium level of education (*p* = 0.55).

### 3.3. MoCA Scoresin Individuals with MCI

A total of 100 subjects with MCI (28 males, 28.0%), with a mean age of 68.52 ± 8.62 years and mean duration of education of 8.24 ± 4.24 years, were tested. The mean MoCA score of the subjects was 24.07 ± 1.57 and the mean MMSE score was 27.41 ± 0.92. The mean total MoCA score and its sub-category scores, according to the sex, age, and education of subjects, are shown in Table 4. The MoCA total score was not significantly affected by sex (F_1,90_ = 0.524, *p* = 0.47, η^2^ = 0.006), but was negatively affected by increased age (F_2,90_ = 5.958, *p* = 0.004, η^2^ = 0.117) and showed a tendency towards higher values as education level increased (F_2,90_ = 2.868, *p* = 0.06, η^2^ = 0.061). Regarding to age, younger adults performed higher MoCA total score compared to older participants (*p* = 0.032) but not compared to adults aged 65–74 years (*p* = 0.148); no significant difference in the MoCA total score was found between ages 65–74 years and >74 years (*p* = 0.51). MANOVA, which was also used to investigate the effects of sex, age, and education on the sub-scale scores of the MoCA, revealed that sex had statistically significant effects on the sub-scores of attention (F_1,90_ = 5.124, *p* = 0.026, η^2^ = 0.077) and orientation (F_1,90_ = 4.070, *p* = 0.047, η^2^ = 0.043), age had a significant effect on the sub-score of visuospatial (F_2,90_ = 4.202, *p* = 0.018, η^2^ = 0.081), and education had significant effects on the sub-scores of visuospatial (F_2,90_ = 6.193, *p* = 0.003, η^2^ = 0.121), language (F_2,90_ = 5.609, *p* = 0.005, η^2^ = 0.111), and abstraction (F_2,90_ = 3.574, *p* = 0.032, η^2^ = 0.074). In particular, younger adults performed better in the visuospatial ability compared to older participants (*p* = 0.047) but not compared to adults aged 65–74 years (*p* = 0.08); no significant difference was found between ages 65–74 years and >74 years (*p* = 0.90). Furthermore, in the cognitive abilities of visuospatial, language and abstraction, higher level of education performed better from those with low (*p* = 0.001, *p* = 0.004 and *p* = 0.004, respectively) but not with medium (*p* = 0.09, *p* = 0.68 and *p* = 0.37, respectively) level. No significant differences in these abilities were observed between MCI subjects with low and medium level of education (*p* = 0.35, *p* = 0.07 and *p* = 0.37, respectively).

The MoCA score, before applying the one point correction for low and medium education groups, was 22.86 ± 1.39 (low education), 23.15 ± 1.88 (medium education), 24.69 ± 1.49 (high education). Higher level of education performed better MoCA total score than those with low (*p* < 0.001) and medium (*p* = 0.01) level; no significant difference was observed between low and medium level of education (*p* = 0.71).

### 3.4. MoCA Scoresin Individuals with Dementia

A total of 119 subjects with dementia (49 males, 41.2%), with a mean age of 72.92 ± 7.54 years and mean duration of education of 6.49 ± 4.03 years, were also tested. The mean MoCA score of the subjects was 16.69 ± 3.38 and the mean MMSE score was 21.76 ± 3.64. The mean total MoCA score and its sub-category scores, according to the sex, age, and education of subjects, are shown in Table 5. The MoCA total score was not affected significantly by sex (F_1,109_ = 0.600, *p* = 0.44, η^2^ = 0.005), age (F_2,109_ = 0.242, *p* = 0.78, η^2^ = 0.004), or education (F_2,109_ = 0.034, *p* = 0.97, η^2^ = 0.001). MANOVA, which was also used to investigate the effects of sex, age, and education on the sub-scale scores of the MoCA, revealed that sex had a statistically significant effect on the sub-score of delayed recall (F_1,108_ = 4.617, *p* = 0.03, η^2^ = 0.041), age had significant effects on the sub-scores of naming (F_2,108_ = 3.361, *p* = 0.04, η^2^ = 0.059) and orientation (F_2,108_ = 3.153, *p* = 0.047, η^2^ = 0.055), and education had a significant effect on the sub-score of visuospatial (F_2,108_ = 3.975, *p* = 0.02, η^2^ = 0.069). In particular, younger adults performed better in the cognitive abilities of naming and orientation compared to older participants (*p* = 0.043 and *p* = 0.016, respectively) but not compared to adults aged 65–74 years (*p* = 0.40 and *p* = 0.39, respectively); no significant difference was found between ages 65–74 years and >74 years (*p* = 0.442 and *p* = 0.08, respectively). Furthermore, regarding the cognitive ability of visuospatial, higher level of education performed better from those with low (*p* = 0.038) but not with medium (*p* = 0.31) level; no significant difference was observed between demented patients with low and medium levels of education (*p* = 0.81).

The MoCA score, before applying the one point correction for low and medium education groups, was 16.78 ± 3.49 (low education), 16.48 ± 2.41 (medium education), 16.45 ± 4.41 (high education), *p* = 0.74.

### 3.5. Discrimination Ability of the MoCA Score Regarding Healthy Subjects and MCI

ROC analysis was performed, in order to evaluate the ability of MoCA total score to discriminate healthy subjects from individuals with MCI (Table 6). The area under the ROC curve for the discriminant potential of the MoCA score for MCI was 0.968 (95% confidence interval (CI) = 0.945–0.991, *p* < 0.001), which indicates an excellent discrimination significance. The optimal cut-off point of 25 for the MoCA total score, which was determined to classify healthy subjects from individuals with MCI, yielded a high sensitivity of 83.0% and a very high specificity of 96.9%. The overall correct classification, according to the MoCA total score, was 88.4%. Cutoff scores for MCI versus healthy subjects for different education levels are presented in Table 6.

### 3.6. Discrimination Ability of the MoCA Score Regarding MCI and Dementia

ROC analysis was performed, in order to evaluate the ability of the MoCA total score to discriminate subjects with MCI from demented subjects (Table 6). The area under the ROC curve for the discriminant potential of the MoCA score for dementia was 0.989 (95% CI = 0.979–1.000, *p* < 0.001), which indicates an excellent discrimination significance. The optimal cut-off point of 21 for the MoCA total score, which was determined to classify subjects with MCI from demented subjects, yielded a very high sensitivity of 96.6% and a very high specificity of 95.0%. The overall correct classification, according to the MoCA total score, was 95.9%. Cutoff scores for subjects with MCI versus demented subjects for different education levels are presented in Table 6.

### 3.7. Discrimination Ability of MoCA Score Regarding Healthy Subjects and Dementia

ROC analysis was performed, in order to evaluate the ability of MoCA total score to discriminate healthy subjects from demented subjects. The area under the ROC curve for the discriminant potential of the MoCA score for dementia was 1.000 (*p* < 0.001), which indicates a perfect discrimination significance. The optimal cut-off point of 24 for the MoCA total score, which was determined to classify healthy from demented subjects, yielded excellent sensitivity and specificity of 100.0%. The overall correct classification, according to the MoCA total score, was 100.0%. Identical results for the discrimination between healthy subjects and demented subjects were found for different education levels.

## 4. Discussion

The design of this study focused on the influence of specific demographic aspects on the performance of the MoCA performance in healthy adults over 50, individuals with MCI, and demented patients in the Greek version of the MoCA and its sub-domains. Additional information was included about the investigation of the diagnostic accuracy of the screening test of the MoCA in a heterogeneous sample of adults.

Our study aimed to add more clinical value in the previous studies [13,14] conducted in Greece about MoCA’s utility in clinical practice, by providing data mainly from a rural population of Northern eastern Greece, recruiting the study under a strict diagnostic protocol.

The results indicated that specific MoCA sub-domains scores were significantly influenced by education in the three groups, and by age and sex in the MCI and dementia groups. In the group of healthy adults, a significant statistical association was found between educational level and cognitive sub-tests of language and abstraction, while sex affected the sub-domain of delayed recall (Table 3). Healthy participants with a higher level of education performed better than those with low level, in the cognitive abilities of language and abstraction. Educational level affected the cognitive sub-tests of visuospatial ability, language, and abstraction in individuals with MCI. Moreover, age affected the MoCA total score and the sub-domain of visuospatial ability. Finally, a significant effect was found between sex and the cognitive sub-tests of attention and orientation (Table 4). MCI individuals with higher level of education performed better than those with low level, in the subdomains of visuospatial ability, language and abstraction. In the group of demented patients, the educational level affected visuospatial naming ability. Significant statistical effects were found between age and the sub-domains of naming and orientation, while sex affected only the cognitive domain of delayed recall (Table 5). Demented patients with high level of education performed better than those with low level of education in the cognitive subdomain of visuospatial ability.

Studies in populations with heterogeneity in socioeconomic background imply the need for normative values, in order to maximize the diagnostic accuracy. In this study, there were mainly included aged participants from rural areas of northern-eastern Greece, who do not have easy access to primary health services and medical resources, most of whom lack of family support, have low income, and fall into the ≤6 years of education category (one of the three educational categories of the sample). The average formal education in Greece has been determined as nine (9) years since 1985, which can explain the discrepancies in education. Moreover, the economic life in these areas is mainly based on agriculture and livestock, indicating low economic status and higher distance from sources of learning and education. These characteristics can influence the quality-of-life of and decisions made by the aged people. Extensive interventions are vitally important, in order to improve the outpatient care system for aged people living in rural areas [39]. We included individuals with low levels of education (≤6 years of formal education) and used the 1-point correction, as suggested in the original study [11]; however, in the literature, this decision has been debated as insufficient to compensate for educational differences [40]. Lack of an effect of education on the MoCA score is likely due to the one-point correction. With the suggested 1-point education correction, healthy and MCI participants in the present sample scored higher, indicating that the 1-point correction may be adequate in similar groups. Using the 1-point correction has been found appropriate for positively affecting the reliability of the MoCA in such samples.

The effect of age on performance in the total MoCAscore was statistically significant, a finding that is consistent with previous studies [13,15,17,21,22,23,24,26,27,28,29,30,31]. In healthy group, younger adults performed better in the visuospatial ability, which gradually decreases as the age increases. The same finding is observed in MCI group, in which younger adults also performed higher in the MoCA total score than the older participants. In the dementia group, younger adults performed better than the older ones in the cognitive abilities of naming and orientation, where we observed a gradual decrease.

The influence of age could be a matter of the research design. This is the reason why Borland et al. [28] suggested in their study that when we investigate cognitive impairment we should include adults over the age of 65. Moreover, the interpretation of cognitive tests should depend on population-based normative data, which is suitable for the population on which it is being used. In the present study, we included age groups over 50 years. Salthouse et al. [41,42,43] have reported that many cognitive abilities (i.e., reasoning, language, memory, and speed) and executive functions decline with age. Many systems of memory are affected in relation to the age, such as semantic memory [44], episodic memory [45,46], and working memory [47]. The changes in cognitive abilities can be interpreted by normative aging processes [48]. In the study of Burke et al. there is an explanation for the effect of age on visuospatial ability, which seemed to be related to the functionality of working memory and attentional processes [49].

Finally, sex had a significant effect on sub-categories of delayed recall, attention, and orientation. Women performed better in delayed recall and orientation in the dementia and healthy group, while men excelled in attention skills in the MCI group. Similar results have beenobserved in the study of Mittal et al. [50], in which they used the MoCA test in order to assess gender-based variations in cognitive function. The influence of sex in screening tests has been controversial in the cited literature. Some studies havesuggested the importance of the variable of sex [13,15,27,28,29], while other studies have indicated that sex does not affect theMoCA score [14,17,22,24,26,30]. They have all investigated the influence of sex on the total score of the MoCA test. Only Santangelo et al. [24] investigated the effect of sex on each sub-domain of the MoCA test, highlighting the relationships of the cognitive abilities of attention and memory with the variable of sex. The sex differences we observed can be interpreted both within the socio-cultural context and in the perception regarding gender diversity.

The results indicated lack of demographic differences, except of age on MCI group, on the total MoCA score, a finding that supports the high reliability of the MoCA test in terms of its diagnostic ability.

The study and the expansion of the discussion in the individual cognitive areas examined by the test provided useful information both for the diagnostic ability of the test and for the overall profile of the examinees at the cognitive and executive level. In clinical practice, this information can be used for the configuration of the treatment and for the design of the cognitive interventions that should be provided.

In the present study, optimal MoCA cutoff points were developed, based on educational level (which was divided into three groups; see Table 6). The MoCA presented high levels of sensitivity and specificity for the detection of MCI and AD in the three educational sub-groups. These scores were consistent with the original validation study of Nasreddine; while, in the more recent study of Carson, Leachand Murphy [51], lower points have been proposed.

According to previous normative values in Greece [13,14], this study presented common elements with the second one, in the design of the study protocol with the main difference that they did not include a strictly healthy control group, but individuals with subjective cognitive decline. If we consider that both groups are in the context of normal aging, then we can state that we both proposed 26 as a cut off score for discriminating highly educated healthy individuals from MCI individuals, whilst for low and middle educated we proposed higher cut off scores (25) than the respective 23 and 26. With regard to the diagnostic ability between healthy and demented patients, we proposed 24 as a cut off score for all educational levels, while their study proposed 20 for the low educated and 23 for middle and high educated participants. This difference is due to the fact that in their own research a statistically significant effect was found on the overall score of the MoCA test, while in ours this difference did not emerge after the choice to use the 1 point correction for participants with low and medium levels of education.

A main contribution of this study is the introduction of a new proposed cut-off score for Greek population, which distinguishes MCI with demented patients. Normal aging, MCI and dementia represent a continuum of cognitive states in the elderly individuals. The cutoff scores indicate points of transition to this continuum. O’Caoimh [52] noted the necessity of using instruments to identify MCI and monitor progression to dementia. They focused on separating MCI from dementia. Moreover, in the study of Trzepacz [53], authors noticed that the research around MoCA has focused on MCI defined more consistently with what is now considered late MCI. Τhis demonstrates the need to increase capacity to capture the full range of MCI cases. They found MoCA ≥17 as the cutoff between MCI and dementia. The results of this study reaffirmed the high sensitivity of the MoCA but suggested a higher cut-off (21) in this setting.

The results of the present study support the findings of previous research, regarding the need for normative data adjusted to individual sociodemographic characteristics. These findings may enhance the clinical use of the MoCA test.

### Limitations and Strengths

The strengths of this study concern the existence of three groups (healthy, MCI, demented), thus increasing the usefulness of the results in a larger sample of the population, especially by health care professionals. A strict protocol was followed for the recruitment of the sample, with individuals undergoing a thorough medical and neurological evaluation, including structural brain imaging. This procedure decreased the number of participants, especially for the healthy group, which is considered to be a major limitation of the study, as larger samples could decrease the risk of sampling errors. The sample comprised the population in the regional area of north-eastern Greece. The generalizability of the results in other ethnic groups and in age groups less than 50 years might be questioned. Another limitation of our study is the small sample size in some groups of subjects in MANOVA.

**Figure 1:**
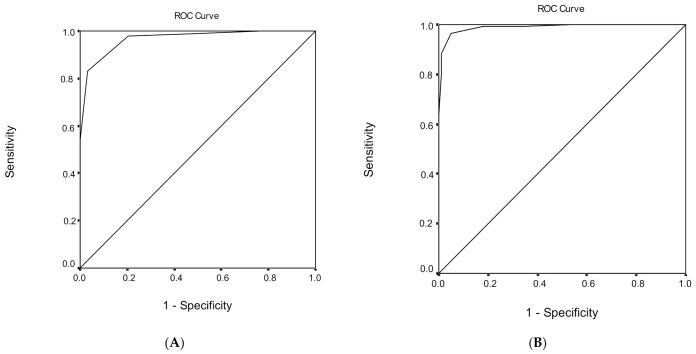
Receiver operating characteristic (ROC) curve analysis of the Montreal Cognitive Assessment (MoCA) to discriminate between (**A**) healthy subjects and mild cognitive impairment (MCI) and (**B**) mild cognitive impairment (MCI) and dementia.

**Figure 2:**
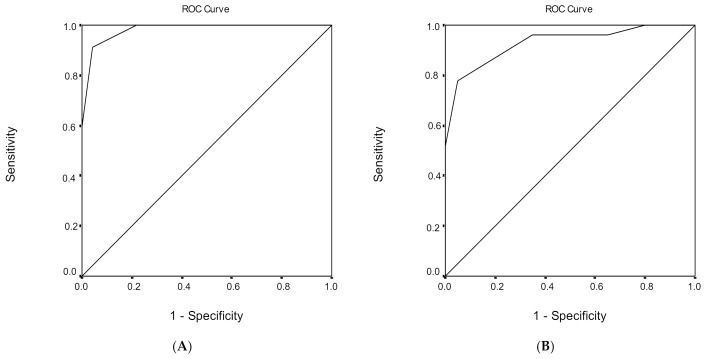

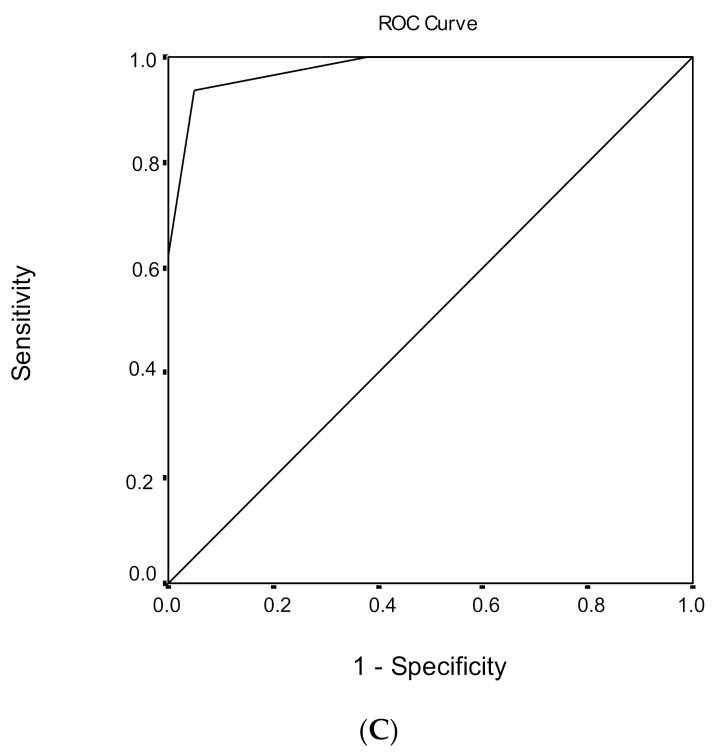
Receiver operating characteristic (ROC) curve analysis of the Montreal Cognitive Assessment (MoCA) to discriminate between healthy subjects and mild cognitive impairment (MCI) under (**A**) low, (**B**) medium, and (**C**) high educational level.

**Figure 3:**
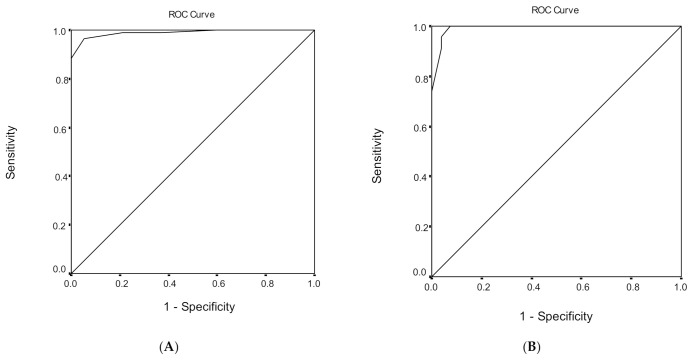

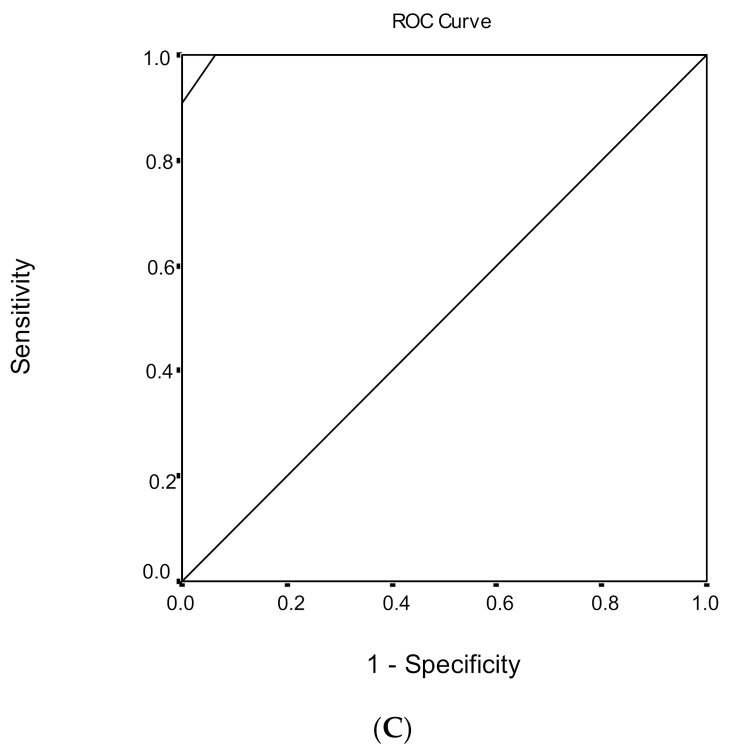
Receiver operating characteristic (ROC) curve analysis of the Montreal Cognitive Assessment (MoCA) to discriminate between mild cognitive impairment (MCI) and dementia under (**A**) low, (**B**) medium, and (**C**) high educational level.

The findings of the study established three separate cut-off scores (Figure 1, Figure 2 and Figure 3) for MCI and dementia, providing more information about the diagnostic validity of the MoCA. Further research should focus on neuropsychological testing as a predictor of cognitive decline [54], in order to provide clinicians with more accurate estimates and assessments. It is important to test the efficacy of measures commonly used in clinical practice and their contribution to the diagnosis of dementia. The study of Ventura et al. [55] reached a similar result, regarding the use of a test which is generalizable and has good diagnostic capability. Clinical data from other neurocognitive disorders could offer important research and clinical information.

## Figures and Tables

**Table 1:** Overview of international studies on normative data (listed by year of publication).

Researchers	Language of Montreal Cognitive Assessment Test	Participant’s Range of Age (Years)	Participant Groups	Effects of Education, Age, Sex	Suggested Cut-Off Scores for the MoCA
Lee JY. et al., 2008 [10]	Korean	>65	Normal Control (NC) Mild Cognitive Impairment (MCI) Alzheimer’s Disease (AD)	(+) education	22/23
Rahman T.T.A. and El Gaafry M. M., 2009 [15]	Arabic	60–83	Normal Mild Cognitive Impairment	(+) education (+) age (+) sex	They used the suggested cut-off score (26) of the original study
Fujiwara Y. et al., 2010 [16]	Japanese	Means 76.4, 77.3, 77.5	Cognitive normal (NC) Mild Cognitive Impairment (MCI) Mild Alzheimer’s Disease (AD)	(−) education (−) age (−) sex	25/26: MCI from Normal Control 25/26: AD from Normal Control
Freitas S. et al., 2011 [17]	Portuguese	>25	Healthy	(+) education (+) age (−) sex	Adjusted normative values
Lu J. et al., 2011 [18]	Chinese	>65	Normal Control (NC) Mild Cognitive Impairment (MCI) Dementia	(+) education (+) age (+) sex (+) urban or rural residence	13/14: Illiterate individuals 19/20: 1–6 years of education 24/25: 7 or moreyears of education
Rosseti et al., 2011 [19]	English (multiethnic study)	18–85	Normal (general population)	(+) education (+) age	Adjusted normative values
Magierska J. et al., 2012 [20]	Polish	Means 76.3, 74.2, 71.4	Cognitively Intact Controls Mild Cognitive Impairment Dementia	(+) education (+) age (−) sex	24 (screening MCI) 19 (screening dementia)
Kenny R. et al., 2013 [21]	Irish	>50	Healthy (not demented)	(+) education (+) age	Adjusted normative values
Narazaki K. et al.,2013 [22]	Japanese	>65	Healthy	(+) education (+) age (−) sex	Adjusted normative values
Malek-Ahmadi M. et al., 2015 [23]	English	70–99	Healthy	(+) education (+) age	Adjusted normative values 25.03 (mean score)
Santangelo G. et al., 2015 [24]	Italian	21–95	Healthy	(+) education (+) age (−) sex	15.5
Ng TP. et al., 2015 [25]	Chinese English	Means 70.8, 69.5, 62.0 58.7	Normal cognition Mild Cognitive Impairment	(+) education	21/22: No education 22/23: 1–6 years of education 27/28: >6 years of education
Kopecek M.et al, 2016 [26]	Czech	>60	Healthy	(+) education (+) age (−) sex	Adjusted for education and age
Konstantopoulos K. et al., 2016 [13]	Greek	>20	Healthy Parkinsonian dementia	(+) education (+) age (+) sex	21 (detecting Parkinsonian Dementia)
Larouche E. et al., 2016 [27]	French	41–98	Healthy	(+) education (+) age (+) sex	Regression-based norms
Borland E. et al., 2017 [28]	Swedish	65–85	Healthy	(+) education (+) age (+) sex	21–25 (lower education) 24–26 (higher education)
Thomann A. et al., 2018 [29]	German	>65	Healthy	(+) education (+) age (+) sex	Adjusted normative values
Apolinario D. et al., 2018 [30]	Brazilian	50–90	Healthy	(+) education (+) age (−) sex	Adjusting to education and age
Cesar K. et al., 2019 [31]	Brazilian	>60	Cognitive normal Cognitive Impaired no dementia Dementia	(+) education (+) age (+) sex (except from dementia group)	15 (distinguish cognitive normal from dementia) 19 (distinguish cognitive normal from cognitive impaired/no dementia)
Poptsi E. et al., 2019 [14]	Greek	>60	Subjective Cognitive Decline (SCD) Mild Cognitive Impairment (MCI) Dementia	(+) education (−) age (−) sex	23–26 (to distinguish adults with subjective cognitive decline from MCI) 20 (to distinguish adults with subjective cognitive decline from dementia)

**Table 2:** Comparison of demographic and clinical characteristics between the three groups.

	Healthy Subjects	MCI	Dementia	*p* Value
*n*	64	100	119	
Male sex	14 (21.9)	28 (28.0)	49 (41.2)	0.016
Age, years	66.73 ± 7.60	68.52 ± 8.62	72.92 ± 7.54	<0.001
Age category				<0.001
50–64 years	25 (39.1)	27 (27.0)	16 (13.4)	
65–74 years	31 (48.4)	51 (51.0)	55 (46.2)	
≥75 years	8 (12.5)	22 (22.0)	48 (40.3)	
Education level				<0.001
Low	23 (35.9)	57 (57.0)	85 (71.4)	
Medium	20 (31.3)	27 (27.0)	116 (19.3)	
High	21 (32.8)	16 (16.0)	11 (9.2)	
Mini Mental State Examination score	29.11 ± 0.69	27.41 ± 0.92	21.76 ± 3.64	<0.001
Total MOCA score	27.58 ± 1.26	24.07 ± 1.57	16.69 ± 3.38	<0.001
Visuospatial	4.38 ± 0.68	3.59 ± 0.88	1.77 ± 1.13	<0.001
Naming	2.97 ± 0.18	2.93 ± 0.26	2.53 ± 0.58	<0.001
Memory	4.97 ± 0.18	4.90 ± 0.33	3.59 ± 0.71	<0.001
Attention	5.77 ± 0.46	5.20 ± 0.94	4.10 ± 1.55	<0.001
Language	2.44 ± 0.61	1.84 ± 0.65	0.76 ± 0.63	<0.001
Abstraction	1.70 ± 0.46	1.47 ± 0.56	1.11 ± 0.55	<0.001
Delayed recall	3.89 ± 0.86	2.45 ± 1.42	0.91 ± 1.26	<0.001
Orientation	5.97 ± 0.17	5.92 ± 0.27	4.68 ± 1.10	<0.001

Qualitative variables are expressed as frequencies and percentages (%) and quantitative variables are expressed as mean values ± standard deviation (SD).

**Table 3:** Effects of characteristics of subjects on MoCA total score and its sub-domains in healthy subjects.

	Sex				Age			Education Level			
	Males	Females	*p* Value	50–64 Years	65–74 Years	>74 Years	*p* Value	Low	Medium	High	*p* Value
MOCA (total)	27.43 ± 1.28	27.62 ± 1.26	0.87	27.68 ± 1.18	27.48 ± 1.34	27.63 ± 1.30	0.86	27.65 ± 1.37	27.25 ± 1.41	27.81 ± 0.93	0.35
Visuospatial	4.50 ± 0.76	4.34 ± 0.66	0.06	4.44 ± 0.65	4.42 ± 0.67	4.00 ± 0.76	0.035	4.43 ± 0.59	4.35 ± 0.75	4.33 ± 0.73	0.10
Naming	3.00 ± 0.00	2.96 ± 0.20	0.77	3.00 ± 0.00	2.94 ± 0.25	3.00 ± 0.00	0.54	2.91 ± 0.29	3.00 ± 0.00	3.00 ± 0.00	0.73
Memory	5.00 ± 0.00	4.96 ± 0.20	0.52	5.00 ± 0.00	4.97 ± 0.18	4.88 ± 0.35	0.43	4.96 ± 0.21	4.95 ± 0.22	5.00 ± 0.00	0.61
Attention	5.86 ± 0.36	5.74 ± 0.49	0.23	5.76 ± 0.44	5.74 ± 0.51	5.88 ± 0.35	0.99	5.74 ± 0.54	5.75 ± 0.44	5.81 ± 0.40	0.66
Language	2.64 ± 0.50	2.38 ± 0.64	0.77	2.36 ± 0.57	2.42 ± 0.67	2.75 ± 0.46	0.13	2.13 ± 0.63	2.40 ± 0.60	2.81 ± 0.40	0.019
Abstraction	1.79 ± 0.43	1.68 ± 0.47	0.74	1.68 ± 0.48	1.74 ± 0.44	1.63 ± 0.52	0.47	1.48 ± 0.51	1.75 ± 0.44	1.90 ± 0.30	0.020
Delayed recall	3.50 ± 0.76	4.00 ± 0.86	0.016	4.08 ± 0.95	3.77 ± 0.84	3.75 ± 0.46	0.33	3.96 ± 0.77	3.65 ± 0.93	4.05 ± 0.86	0.35
Orientation	5.93 ± 0.27	5.98 ± 0.14	0.21	5.96 ± 0.20	5.97 ± 0.18	6.00 ± 0.00	0.72	6.00 ± 0.00	5.95 ± 0.22	5.95 ± 0.22	0.96

Data are expressed as mean value ± standard deviation (SD).

**Table 4:** Effects of characteristics of subjects on MoCA total score and its sub-domains in subjects with MCI.

	Sex				Age			Education Level			
	Males	Females	*p* Value	50–64 Years	65–74 Years	>74 Years	*p* Value	Low	Medium	High	*p* Value
MOCA (total)	24.00 ± 1.61	24.10 ± 1.56	0.47	24.67 ± 1.57	23.98 ± 1.38	23.55 ± 1.79	0.004	23.86 ± 1.39	24.15 ± 1.88	24.69 ± 1.49	0.06
Visuospatial	3.54 ± 0.84	3.61 ± 0.90	0.43	4.11 ± 0.75	3.45 ± 0.90	3.27 ± 0.70	0.018	3.30 ± 0.80	3.78 ± 0.85	4.31 ± 0.70	0.003
Naming	2.89 ± 0.31	2.94 ± 0.23	0.95	2.93 ± 0.27	2.96 ± 0.20	2.86 ± 0.35	0.16	2.89 ± 0.31	2.96 ± 0.19	3.00 ± 0.00	0.12
Memory	4.93 ± 0.26	4.89 ± 0.36	0.26	4.93 ± 0.27	4.94 ± 0.24	4.77 ± 0.53	0.16	4.88 ± 0.38	4.93 ± 0.27	4.94 ± 0.25	0.54
Attention	5.54 ± 0.74	5.07 ± 0.98	0.026	5.22 ± 0.80	5.02 ± 1.05	5.59 ± 0.73	0.48	5.25 ± 0.91	5.04 ± 1.09	5.31 ± 0.79	0.80
Language	1.79 ± 0.79	1.86 ± 0.59	0.96	1.89 ± 0.70	1.90 ± 0.67	1.64 ± 0.49	0.18	1.65 ± 0.55	2.04 ± 0.71	2.19 ± 0.66	0.005
Abstraction	1.50 ± 0.58	1.46 ± 0.56	0.36	1.52 ± 0.51	1.55 ± 0.58	1.23 ± 0.53	0.06	1.32 ± 0.54	1.59 ± 0.57	1.81 ± 0.40	0.032
Delayed recall	2.36 ± 1.39	2.49 ± 1.43	0.72	2.59 ± 1.53	2.43 ± 1.42	2.32 ± 1.32	0.22	2.51 ± 1.40	2.48 ± 1.40	2.19 ± 1.56	0.36
Orientation	5.82 ± 0.39	5.96 ± 0.20	0.047	5.93 ± 0.27	5.94 ± 0.24	5.86 ± 0.35	0.99	5.93 ± 0.26	5.93 ± 0.27	5.88 ± 0.34	0.69

Data are expressed as mean value ± standard deviation (SD).

**Table 5:** Effects of characteristics of subjects on MoCA total score and its sub-domains in demented patients.

	Sex				Age			Education Level			
	Males	Females	*p* Value	50–64 Years	65–74 Years	>74 Years	*p* Value	Low	Medium	High	*p* Value
MOCA (total)	16.35 ± 3.15	16.93 ± 3.54	0.44	16.88 ± 3.86	17.16 ± 3.22	16.08 ± 3.37	0.78	16.78 ± 3.49	16.48 ± 2.41	16.45 ± 4.41	0.97
Visuospatial	1.86 ± 1.27	1.71 ± 1.02	0.97	1.81 ± 1.38	1.89 ± 1.01	1.63 ± 1.18	0.49	1.65 ± 1.00	1.87 ± 1.22	2.55 ± 1.63	0.022
Naming	2.46 ± 0.54	2.59 ± 0.60	0.22	2.80 ± 0.41	2.56 ± 0.60	2.42 ± 0.58	0.038	2.52 ± 0.59	2.57 ± 0.59	2.55 ± 0.52	0.62
Memory	3.53 ± 0.71	3.63 ± 0.71	0.69	3.63 ± 0.89	3.69 ± 0.77	3.46 ± 0.54	0.61	3.59 ± 0.71	3.59 ± 0.71	3.55 ± 0.93	0.94
Attention	4.27 ± 1.47	3.99 ± 1.60	0.18	4.25 ± 1.34	4.09 ± 1.52	4.06 ± 1.67	0.64	4.14 ± 1.60	4.00 ± 1.41	4.00 ± 1.48	0.64
Language	0.84 ± 0.69	0.71 ± 0.59	0.40	0.75 ± 0.58	0.85 ± 0.65	0.67 ± 0.63	0.95	0.74 ± 0.62	0.74 ± 0.54	1.00 ± 0.89	0.35
Abstraction	1.06 ± 0.63	1.14 ± 0.49	0.23	1.25 ± 0.68	1.09 ± 0.59	1.08 ± 0.45	0.65	1.05 ± 0.55	1.26 ± 0.54	1.27 ± 0.47	0.24
Delayed recall	0.53 ± 0.98	1.17 ± 1.37	0.034	0.81 ± 1.22	0.87 ± 1.25	0.98 ± 1.31	0.80	1.01 ± 1.31	0.74 ± 1.05	0.45 ± 1.21	0.75
Orientation	4.61 ± 1.04	4.73 ± 1.14	0.10	5.00 ± 1.21	4.84 ± 1.01	4.40 ± 1.11	0.047	4.71 ± 1.17	4.65 ± 0.78	4.55 ± 1.13	0.43

Data are expressed as mean value ± standard deviation (SD).

**Table 6:** Diagnostic ability of the MoCA.

Education Level	AUC (95% CI)	*p* Value	Cut-Off	Sensitivity (%)	Specificity (%)	PPV (%)	NPV (%)	Overall Agreement	+LR	−LR
Healthy to MCI										
Low	0.982 (0.956–1.000)	<0.001	≤25	91.2	95.7	98.1	81.5	92.5	21.21	0.092
Medium	0.930 (0.858–1.000)	<0.001	≤25	77.8	95.0	95.5	76.0	85.1	15.56	0.234
High	0.979 (0.941–1.000)	<0.001	≤26	93.8	95.2	93.8	95.2	94.6	19.54	0.065
Total sample	0.968 (0.945–0.991)	<0.001	≤25	83.0	96.9	97.6	78.5	88.4	26.77	0.174
MCI to Dementia										
Low	0.989 (0.976–1.000)	<0.001	≤21	96.5	94.7	96.5	94.7	95.7	18.21	0.037
Medium	0.993 (0.977–1.000)	<0.001	≤21	100.0	92.6	92.0	100.0	96.0	13.51	0
High	0.997 (0.986–1.000)	<0.001	≤22	100.0	93.7	91.7	100.0	96.3	15.87	0
Total sample	0.989 (0.979–1.000)	<0.001	≤21	96.6	95	95.8	96.0	95.9	19.32	0.036
Healthy to Dementia										
Low	1.000 (1.000–1.000)	<0.001	≤24	100.0	100.0	100.0	100.0	100.0	-	0
Medium	1.000 (1.000–1.000)	<0.001	≤24	100.0	100.0	100.0	100.0	100.0	-	0
High	1.000 (1.000–1.000)	<0.001	≤24	100.0	100.0	100.0	100.0	100.0	-	0
Total sample	1.000 (1.000–1.000)	<0.001	≤24	100.0	100.0	100.0	100.0	100.0	-	0

AUC, area under the curve; CI, confidence interval; PPV, positive predictive value; NPV, negative predictive value; +LR, positive likelihood ratio; −LR, negative likelihood ratio.

## Data Availability

The data that support the findings of this study are available from the corresponding author upon reasonable request.
